# Harmful Marketing: An Overlooked Social Determinant of Health

**DOI:** 10.1007/s11121-024-01763-x

**Published:** 2025-01-10

**Authors:** Erika Westling, James Gordon, Paul M. Meng, Cassandra A. O’Hara, Brandon Purdum, Andrew C. Bonner, Anthony Biglan

**Affiliations:** 1 Oregon Research Institute, 3800 Sports Way, Springfield, OR 97477 USA; 2 Fordham University, Bronx, USA; 3 University of Hawaii at Mānoa, Honolulu, USA; 4 University of Florida, Gainesville, USA; 5 Littleton, USA; 6 Northeastern University, Boston, USA

**Keywords:** Harmful marketing, Commercial determinants of health, Tobacco, Cigarettes, Alcohol, Processed food, Fossil fuels, Firearms, Guns, Pharmaceuticals, Opioids

## Abstract

This paper reviews evidence about the impact of marketing on ill health. We summarize evidence that marketing practices in six industries (tobacco, alcohol, pharmaceutical, processed food, firearm, and fossil fuel) are causal influences on the occurrence of injury, disease, and premature death. For each industry, we provide a brief overview on the extent of harmful marketing, efforts from each industry to obscure or otherwise conceal the impact of their marketing strategies, and efforts to counter the impact of harmful marketing in these industries. However, considering the ubiquitous belief that regulation is harmful to society, little headway has been made in reducing harmful marketing. We propose the substitution of a public health framework for the currently dominant free market ideology. Doing so would situate harmful marketing as a social determinant of health and consolidate the disparate efforts to regulate marketing of harmful products. Implications for future policy and research efforts are discussed.

**Supplementary Information:**

The online version contains supplementary material available at 10.1007/s11121-024-01763-x.

## Harmful Marketing: An Overlooked Social Determinant of Health

This paper examines the impact of marketing on health in the USA, as commercial determinants of health have received far less attention in the literature on social determinants of health, despite their significant impact (Maani et al., 2020). We review evidence showing that marketing practices in six industries—tobacco, alcohol, pharmaceuticals/opioids, processed food, firearms, and fossil fuels—causally influence the incidence of injury, disease, and premature death. These industries not only promote harmful products but also obscure their negative health impacts to avoid regulatory scrutiny. Although we cannot review all industries that utilize marketing to sell products that harm health (e.g., gambling), we selected these six industries to represent some of the most egregious examples. For these six industries, we describe the epidemiological and economic aspects of associated health outcomes, the marketing strategies of these industries, their resistance to regulation, and efforts to mitigate the effects of harmful marketing. However, in the context of a widespread belief that the regulation of industries is harmful to society, achieving regulation that would decrease the public health impact of these products is challenging. We propose adopting a public health framework in place of the prevailing free market approach. This shift would recognize harmful marketing as a critical social determinant of health and unify various regulatory efforts into a comprehensive public health strategy to control marketing practices that endanger significant portions of the population.

## The Tobacco Industry

### Deaths Due to Use of Cigarettes

In the USA, cigarette smoking continues to be a leading cause of death killing an estimated 480,000 Americans each year. (See Supplement Table 1 for a summary of estimated annual yearly deaths in the USA attributable to cigarettes and other products discussed in this paper, as well as the disproportionate impact of these products on lower income, minoritized communities.)

### The Marketing of Tobacco

Despite clear evidence of the harmful effects of cigarettes, the tobacco industry continues to be a billion-dollar industry. In 2015, the world’s six largest cigarette manufacturers made a profit of more than $62 billion, more money than the combined profits of Coca Cola, Walt Disney, FedEx, Google, Starbucks, and McDonalds (Branston, 2021). It is estimated that the cigarette and smokeless tobacco industries spend $22.5 million every day on advertising efforts. In contrast, the first federally funded anti-smoking campaign spent, in one year, what the tobacco industry spends on advertising and promotion in just three days (Centers for Disease Control and Prevention, 2016).

The tobacco industry capitalizes on human desire for social approval by marketing cigarettes as a path to social acceptance, especially among young people (Biglan, 2020d). In the 1920s, cigarette ads targeted women by linking smoking to weight loss (Truth Initiative, 2017). However, nothing was as successful as the creation of the Marlboro Man, which aimed to convince young men that their masculinity would be enhanced by smoking Marlboros (National Cancer Institute, 2008).

In the USA vs. Philip Morris et al., the court ruled that the marketing of cigarettes to those under 18 was a causal influence on young people’s initiation of smoking. The evidence was reviewed in a monograph of the National Cancer Institute (National Cancer Institute, 2008). It cited numerous correlational studies showing that youth exposure to cigarette ads predicted later uptake of smoking. In addition, favorable attitudes toward smoking and taking up smoking were shown to be strong predictors of later initiation of smoking. The industry’s argument that youth smoking initiation was due to peer and parental influences was refuted by evidence from randomized trials showing that exposure to cigarette advertising increased favorable attitudes toward smoking initiation, which lead to smoking (National Cancer Institute, 2008). These effects were observed regardless of the extent of peer and parental influences.

Recently, electronic cigarettes have been marketed as a safer alternative to combustible cigarettes. There is minimal evidence to support this claim (Truth Initiative, 2021). The Food and Drug Administration (FDA) has regulatory authority and has taken steps to prevent the sale of these products to those under 18. However, more than 2.5 million high school and middle school students reported using e-cigarettes in 2022 (U.S. Food and Drug Administration, 2022).

### Obscuring the Goals and Impact of Tobacco Marketing

The tobacco industry has a long and well-documented history (Oreskes & Conway, 2011) of obscuring the impact of cigarettes on health, as well as the impact its marketing has on young people. When research in the 1950s began to show the impact of smoking on lung cancer, the industry created the Tobacco Industry Research Committee and the Tobacco Institute. Each was organized to cast doubt on the evidence that smoking was harmful and that the industry was marketing to children.

One of the industry’s efforts to obscure the impact of tobacco-related advertising was a campaign that claimed to be designed to influence parents to prevent their children from smoking (Biglan and Cody, 2004). This program was introduced in states that were considering restrictions on marketing to youth. The industry got endorsements from physicians and legislators. However, this campaign was never intended to reach parents. Rather, it was used to reassure political leaders that they did not need to regulate tobacco marketing.

### Efforts to Curb Cigarette Marketing

Significant efforts have been made by the tobacco control movement to reduce harmful marketing practices that lead to smoking. In 1998, the Master Settlement Agreement (MSA) became the largest civil litigation in US history. It also became the turning point for efforts to end tobacco use. The MSA included policies that restricted certain marketing practices, such as ads targeting people under 18, and the use of cartoon characters. It also eliminated outdoor, billboard, and public transit advertising while restricting the use of cigarette brand names on merchandise (Truth Initiative, 2019). As a result of these efforts, as well as increased taxation, widespread smoking cessation programs, and public education, the rate of smoking in the USA has gone from 42% in 1965 to 13.7% in 2018 (American Lung Association, 2022).

The tobacco control movement has become a model in terms of ways to combat harmful marketing practices. Part of the movement’s success has been the establishment of clear evidence showing not only the harm of cigarettes but also how marketing increases the likelihood that an individual will start smoking. The ways this evidence was disseminated and used to influence public policy were vital to the movement’s success (Biglan, 2020d).

## The Alcohol Industry

### Deaths Due to Alcohol Use

See Supplemental Table 1 for attributable deaths due to alcohol use (178,000), as well as details regarding the disproportionate impact of alcohol use on minoritized and low-income individuals. Beyond the burden of alcohol-related mortality, the health and social consequences of excessive alcohol use have been estimated to be more than $223 billion per year (Bouchery et al., 2011).

### Marketing of Alcohol to Young People

Spending on alcohol marketing was projected to increase from $6.7 billion in 2020 to $7.7 billion in 2023, with 30% of that spent on digital advertising (Zenith Media, 2021). Perhaps unsurprisingly, youth exposure to digital and targeted advertising is challenging for independent monitors to track, so this increase complicates efforts to monitor who is seeing alcohol ads. A growing body of research suggests that exposure to alcohol marketing increases the onset and severity of alcohol consumption by youth (e.g., Anderson et al., 2009; Jernigan et al., 2017; Sargent et al., 2020). Much of this work is cross-sectional (e.g., Finan et al., 2020; Henehan et al., 2020), but several systematic reviews, commissioned to assess the association between exposure to alcohol marketing and use by youth, support a causal link (Sargent & Babor, 2020).

### Efforts of the Industry to Conceal Their Impact

Unlike the MSA-imposed regulations on tobacco marketing to youth, there are few restrictions on alcohol marketing in the United States. The alcohol industry has a voluntary, self-regulatory code to not advertise in magazines, in newspapers, on television, on radio, or in digital media in which underage youth comprise more than 28.4% of the audience. The code also promises to not depict excessive drinking or acceptability of drunken behavior in ads and to not associate the consumption of alcohol with status or success (Beer Institute, 2018; Distilled Spirits Council of the United States, 2021; Wine Institute, 2011).

However, there is no independent oversight, monitoring, or enforcement of these industry codes. As a result, they are often disregarded (Federal Trade Commission, 1999; Jernigan et al., 2013). Like the tobacco industry, these industry-imposed self-regulations for alcohol were established to avoid governmental regulation (Babor et al., 2010).

### Limited Impact of Efforts to Reduce Marketing to Youth

In 2002, the Center on Alcohol Marketing and Youth (CAMY) was established to monitor alcohol marketing practices in the USA, with a focus on youth exposure to advertisements in magazines, on television, and on the radio. CAMY analyses found that, between 2001 and 2005, alcohol companies were routinely advertising to audiences with a high number of youths aged between 12 and 20 (Centers for Disease Control and Prevention, 2007). From 2005 to 2012, youth under age 21 were exposed to $15.1 billion of alcohol advertisements that did not comply with the self-imposed industry audience composition guidelines (i.e., over 28.4% of the audience was underage), primarily via cable television (Ross et al., 2016).

A recent study found that, between 2013 and 2018, alcohol ads were appearing more frequently during shows in which 28.4% or more of the audience were under-age viewers (Henehan et al., 2021). In sum, the lack of governmental oversight, regulation, or enforcement of self-imposed industry regulations for alcohol marketing is ineffective tools for reducing exposure of alcohol marketing to youth.

### The Pharmaceutical Industry: Opioids

The pharmaceutical industry has produced many benefits for society. However, it has also engaged in harmful marketing of drugs (Biglan, 2020b). Here, we focus on the marketing of opioids, a particularly egregious example of the harmful marketing done by the pharmaceutical industry. (As documented in Supplemental Table 1, there were more than 100,000 deaths attributed to drug overdoses in 2022.)

### Marketing Supposedly Non-Addictive Opioids

In 1996, Purdue Pharma began marketing OxyContin, a synthetic opioid the company claimed was not addictive. The FDA accepted this claim and, as a result, Purdue Pharma convinced physicians that they could and should be prescribing OxyContin for pain. Purdue aggressively marketed the drug, expanding the number of drug-detail representatives from 318 to 671, as well as the list of physicians it marketed to, from 33,000 to 94,000 (Van Zee, 2009).

OxyContin sales representatives were lavishly compensated for promoting the drug and increasing sales. For example, in addition to an average annual salary of $55,000 USD, sales representatives received annual bonuses of anywhere from $15,000 to $240,000 (Van Zee, 2009). Additional efforts to increase OxyContin prescriptions involved two types of payments to physicians. First, they funded physicians, particularly those who had conducted research on opioids, to present on and promote opioid prescriptions at conferences of physicians. Second, they gave gifts to “top prescribers” in exchange for increasing their OxyContin prescriptions (Chimonas et al., 2010; Mulinari & Ozieranski, 2022; Shakeel et al., 2019; Van Zee, 2009).

Purdue and other companies marketing opioids expanded the market by influencing physicians to prescribe them for patients with non-cancer related pain. As a result, the opioid prescription rate for non-cancer related pain increased from 670,000 in 1997 to 6.2 million in 2002 (Van Zee, 2009). Pharmaceutical firms focused much of their opioid marketing efforts on areas with high rates of worker injuries (Van Zee, 2009). The industry also funded two organizations that pushed to increase rates of pain medication prescription by describing pain as “the fifth vital sign” (Scher et al., 2018). Total profits from the sales of OxyContin have been estimated at $12 to $13 billion USD (Sandler, 2019).

Hadland et al. (2019) demonstrated a critical causal chain from marketing to physicians, to their rate of prescribing, and to the subsequent rate of overdose deaths. The size of investment in marketing opioids within a given county was positively associated with the number of opioid-related deaths. Moreover, the link between marketing and death rate was mediated by prescribing rates. The industry understood that the marketing of drugs to physicians increased their rate of prescribing. But this fact remained largely opaque to the legal and public health communities. As a result, opioid deaths mushroomed (see Supplemental Table 1).

### Efforts to Address the Harm Done by Marketing Opioids

The extent of overdose deaths over a period of more than 20 years represents an abject failure of the public health system. The FDA ignored evidence that the prescribing of opioids was causing death (Kolodny, 2020). Career prosecutors in the US Department of Justice (DOJ) summarized the illegal behavior of Purdue Pharma in 2006, but the DOJ did not pursue prosecution. Senators Hassan and Whitehouse have suggested that the reason for this was Purdue’s outsized influence: the wealthy owners of Purdue Pharma had a bigger influence on DOJ decisions than did the epidemic of opioid-related deaths (Hassan & Whitehouse, 2020).

One major effort to mitigate the harm of marketing opioids and other pharmaceuticals has come in the form of litigation. This litigation has been primarily civil in nature (only rarely have criminal cases been brought related to pharmaceutical marketing). Civil litigation focused on mitigating the impact of harmful pharmaceutical marketing typically begins with an individual suffering an adverse event during or following a course of treatment with a particular drug. However, civil litigation against pharmaceutical companies is difficult for several reasons. Causal claims require a particularly onerous standard of evidence that an individual plaintiff would have difficulty meeting. Few attorneys have the resources to litigate a case against a wealthy pharmaceutical company. This creates an inherently unbalanced and unjust system for mitigating the harm done by pharmaceutical marketing.

### Settlements with Big Pharma

Despite the inherent injustice associated with relying on this mechanism to mitigate harm done by marketing of pharmaceuticals, there have been several large-scale cases that have resulted in costly settlements. While this is in some ways encouraging, it should be noted that without knowing how much a company has profited from the sale of a particular product, it is impossible to know what cost would deter such harmful marketing in the future. These settlements may be viewed as “the cost of doing business.” For example, the best available estimates indicate that Merck secured profits of at least $2 billion *after factoring in the massive penalties and settlements* associated with Vioxx (Wadman, 2007).

Similarly, the Sackler family and their company, Purdue Pharma, paid a record settlement for their harmful marketing of OxyContin. By the best available estimates, the Sackler family still profited by at least $4 billion (Mann & Bebinger, 2022). Their attempt to shield themselves from further liability through a bankruptcy filing was recently overturned by the Supreme Court. Although the court’s decision delays payment to some victims, it ensures that organizations that do harm can no longer avoid accountability by declaring bankruptcy.

## The Processed Food Industry

### Risks of Processed Food

In the USA, two of the top three leading causes of early death are linked to diet: a high body mass index (BMI) and elevated fasting blood sugar levels (Mokdad et al., 2018). Despite progress in other health areas, obesity rates have significantly risen since 1988 (Shields et al., 2011; see Supplemental Table 1). Childhood obesity has also followed this upward trend (The State of Obesity, 2017). Conditions associated with obesity, such as diabetes (5% of healthcare costs) and heart disease (4.3% of healthcare costs), now consume a large share of healthcare spending (Dieleman et al., 2016). (As documented in Supplemental Table 1, more than 300,000 Americans die of cardiometabolic deaths each year.)

### Marketing Processed Food to Children

Children who are exposed to more food-related advertising typically consume more high-calorie, low nutrient food (Gamble & Cotugna, 1999). The World Health Organization (World Health Organization, 2003) found that food marketed to children contributed causatively to childhood obesity. The risk of adult obesity is higher among those who experienced obesity in childhood (Pan et al., 2016).

An American Psychological Association task force concluded that until around age 8, children generally do not realize that advertisements are trying to sell them something, instead, children view ads as a type of public service announcement. According to Linn (2004), advertisers argue that parents are responsible for ensuring protection from the marketing of unhealthy foods. Thus, to advertisers, parents are to blame for childhood obesity.

The food industry is not simply reaching children through large amounts of persuasive advertising of processed food. In fact, the industry has a long history of *designing* foods that pray on our evolved susceptibilities to salt, sugar, and fat (Moss, 2013). At the same time, food manufacturers have made processed foods more convenient and easier to prepare, such that working parents find them more desirable than other more nutritious meals.

Healthy foods can be successfully marketed to children. For example, one study found that simply showing pictures of superheroes eating vegetables across an elementary school cafeteria salad bar nearly doubled vegetable intake among students (Hanks et al., 2016). Yet, the marketing of healthful food makes up less than 5% of total food industry marketing expenditures.

### Food Industry Efforts to Prevent Regulation

Moss (2013) documented that food companies have long known that they contribute to the obesity problem. Moss described an industry conclave in 1999 in which the issue was discussed, and major industry leaders indicated they would not desist from practices that contribute to obesity and disease. Industry practices were and are selected due to their profitability.

### Policy Can Curb the Marketing of Harmful Food

Like other industries that engage in harmful marketing, the food industry has used public relations and lobbying to prevent regulation. For example, sugar manufacturers secretly sponsored the publication of a review of the impact of fat on cardiovascular disease, which downplayed the role of sugar in that disease (Kearns et al., 2016). Thanks to lax government regulations, the food industry largely regulates itself. Much like the tobacco and alcohol industry, such self-regulation has not reduced the food industry’s marketing of harmful products. More than 80% of food marketing is for products in the lowest nutritional bracket (Kunkel et al., 2015).

Regulation can curb the influence marketing has on the consumption of unhealthful foods. In 2016, Chile passed a law that banned cartoon characters from appearing on packaging for cereal and other sugary foods. Chile also banned Kinder Surprise, a chocolate egg that hides a toy inside. This law prohibited the sale of “junk” foods in Chilean schools but has since been overturned following pushback from the food industry. However, unhealthful food sold in Chile now contains black labels warning of high salt, sugar, calories, and fat content. As a result of this legislation, food companies have changed their ingredients to avoid these labels.

## The Firearm Industry

### How The Firearm Industry Markets Its Products

Over the past 70 years, despite an increasing number of deaths by firearms—48,204 in 2022 (see Supplemental Table 1). American gun marketing has shifted from a focus on sportsmen and hunting to an emphasis on home protection and self-defense. The pivot to self-protection was prompted by the upswing in gun sales that followed the 1992 Los Angeles riot (Yamane et al., 2020). The gun industry and advocacy groups like the National Rifle Association (NRA) (n.d.) utilize fear-based themes an approach that has been particularly effective among women (Hagerty, 2012). Gun marketing is also bolstered by the proliferation of shooting ranges as sources of entertainment and community activity centers (Weeks, 2013), as well as more targeted social media advertising designed to improve public sentiment and grow the industry’s reach (Jordan et al., 2020). Gun marketers also target children (Violence Policy Center, 2016).

### How the Firearm Industry Obscures the Harm of Firearm Marketing

Like other industries, the firearm industry dedicates considerable effort to cultivating positive public sentiment and policies that support the sale of its products. Gun industry advocacy is largely managed by two organizations: the National Shooting Sports Foundation (NSSF) and, more notably, the NRA (NRA, n.d.).

The NRA and affiliated organizations spend more money lobbying for the promotion of pro-gun policies than the entire gun-control lobby. They aggressively target state and federal gun control proposals as threats to gun rights and gun ownership (Plumer, 2012). By emphasizing gun control legislation as an attack on second Amendment and government overreach, the NRA and others have cultivated enough ongoing public support and sentiment to stop countless common-sense gun laws and stymie gun violence research across the country (Biglan, 2020e).

Additionally, the NRA and other pro-gun groups often utilize emergency events like natural disasters, terrorist attacks, and social disorder to build fear and tout the importance of guns and self-protection during a crisis (Mascia, 2020). By framing guns as essential and gun control as an existential threat to basic rights, firearms advocacy groups have effectively blurred public perception of gun marketing, gun violence, and its consequences for society at large.

### Efforts to Curb Harmful Firearm Marketing

Over the years, many efforts have been made to reduce the harmful impact of firearm marketing. Yet, most of these efforts have failed, largely due to the significant political influence of powerful gun advocacy groups like the NRA. Such groups have grown and remained strong by continually stoking fears among a certain population that the government is coming to take away their second Amendment rights.

### The Fossil Fuel Industry

Fossil fuels have made an enormous contribution to human wellbeing over the past 200 years. Thanks to the availability of these fuels, the world has experienced unprecedented growth in wealth and wellbeing. Fossil fuels made possible railroads, airplanes, automobiles, and electricity. However, there is overwhelming evidence that this boon to human wellbeing has also resulted in an unprecedented increase in CO_2_, which is causing catastrophic climate change. Here is a summary of the harm that is already well underway. According to the National Oceanic and Atmospheric Administration (NOAA), we are experiencing more severe and more frequent storms, wildfires, flooding, and droughts. In 2020 alone, there were 22 disasters in the USA that cost more than a billion dollars to address. This is six more than the previous record, set in 2017 (National Oceanic and Atmospheric Association, 2021).

Sea levels have already increased nine inches since the late nineteenth century. Forty percent of the US population lives in coastal areas that are already being hit with increased flooding, storm surges, and saltwater intrusion. The cost of this is estimated to be $400 billion over the next 20 years (Bertrand, 2021). As CO_2_ levels rise, the oceans become more acidic; ocean acidity has increased 30 percent over the past 150 years and will continue (Bertrand, 2021). This is harming shellfish and coral reefs and will reduce the supply of seafood.

The Intergovernmental Panel on Climate Change predicts that the Earth’s temperature could rise between 2.5°F and 10°F. July of 2024 was the hottest month on record for in NOAA’s 175-year record and was the 14th consecutive month of record-high global temperatures (National Oceanic and Atmospheric Association, 2021). Our current problems will be nothing compared to what happens with another five-degrees of warming.

In addition to the combustion of fossil fuels causing climate change, the products of the fossil fuel industry have other environmental effects. These include two plastic garbage patches in the Pacific Ocean that are twice the size of Texas (The Ocean Clean Up, n.d.), and microscopic amounts of plastic in our bodies (Parker, 2022). Eight million people around the world died because of fossil fuel pollution in 2018 (Harvard TH Chan, 2021).

### Industry Efforts to Protect Its Investments

Like any industry, the practices of the fossil fuel industry have been selected by their economic consequences. The creation, marketing, and sales of fossil fuel products have been highly remunerative for the coal and oil industry. And like other industries, the fossil fuel industry has evolved its lobbying and public relations practices to protect its interests. When reports began suggesting that fossil fuel emissions could be increasing the Earth’s temperature, the industry took steps to convince the public and policymakers that climate change was not happening, and that fossil fuel use was not warming the planet (Supran et al., 2023).

The industry has good reason to be concerned. If the increase in Earth’s temperature is to be prevented from rising more than 3.6°F above preindustrial levels, about one third of oil reserves, half of gas reserves, and over 80% of current coal reserves would have to remain unused (McGlade & Ekins, 2015). Thus, the companies that own these assets, which have been valued at $33 trillion (Ryan, 2016), would be unable to sell them. Here are some of the ways the oil and gas industry has worked to convince the public and policymakers that climate change is not happening. Like the tobacco industry, this industry has promoted doubt about the scientific evidence that climate change is happening and is human caused (Taylor, 2023).

As one example, a researcher, Wei-Hock “Willie” Soon, an aerospace engineer at the Harvard-Smithsonian Center for Astrophysics, was funded by the fossil fuel industry and lobby (e.g., Exxon Mobil, the industry-funded American Petroleum Institute) to do research that claimed to show that human emissions were not causing climate change—a clear conflict of interest. Soon did not disclose his source of funding, and the Smithsonian also did not disclose his source of funding, enabling this lack of transparency (Goldenberg, 2015).

The fossil fuel industry has actively funded and created organizations, such as the “Advancement of Sound Science Coalition” to spread misinformation, for example by distributing misleading educational materials to schools (Office of the Attorney General for the District of Columbia, 2020). Another fossil fuel lobbying group, the Western States Petroleum Association, has created numerous other organizations to oppose policies aimed at reducing carbon emissions, such as opposition to carbon taxes.

As concerns about fossil fuels have grown, the industry has begun to publicize their support for reducing fossil fuels. However, a Congressional analysis of the industry’s lobbying and production indicated that the companies are increasing their marketing of fossil fuels and spending only a tiny fraction of its lobbying efforts on the promotion of fossil fuel reduction (U. S. Senate Committee of The Budget., 2024). This lobbying appears to be for the purpose of showing that people are doing something about the problem, even as the industry continues to increase its emissions.

Some major financial management organizations have begun to divest themselves from fossil fuel companies. In response, a few states that are heavily influenced by the fossil fuel industry have implemented laws to prohibit companies from doing business with firms that are cutting investments in fossil fuels (Ariza & Buchele, 2022). In sum, the fossil fuel industry continues to market products that are contributing to climate catastrophe while concealing its role in resisting the changes that are needed.

## Discussion

Each industry reviewed here is engaging in marketing practices that contribute to disease, injury, or premature death. As shown in Supplemental Table 1, an estimate of the number of Americans who die each year as a result of marketing these products is well over one million. When seen in this light, harmful marketing should be considered a social determinant of ill health. Reducing such marketing should be a top priority for public health research and practice.

In each case, these industries are engaging in marketing and public relations practices designed to obscure the impact of their product on disability and death, and to prevent regulation that might reduce their profits. Efforts are underway to stop these harmful practices, but they have had limited success, largely because of the skill and resources these industries put toward preventing regulation of their practices (Biglan, 2020c).

The fact that excess deaths continue to occur and are in some cases increasing because of these practices constitutes empirical evidence that our society does not have policies and practices effective at preventing or ameliorating these harms. On that basis, we describe the steps we believe are needed.

### A Registry of Marketing-Related Deaths

In the same way that our public health system monitors deaths due to chronic and infectious illness and injuries, we need a system that provides annual data on deaths attributable to marketing. For example, a study by Pierce et al. (Pierce et al., 1999) estimated that 34% of new smokers begin smoking as a result the advertising and promotion of cigarettes. Based on the number of new smokers who began smoking Camel or Marlboro cigarettes between 1988 and 1998 and the proportion of smokers who die due to smoking, the study estimated that 520,000 people would die due to Camel marketing and 300,000 would die due to Marlboro marketing (Pierce et al., 1999).

Having annual estimates of the deaths due to the marketing of various products would drive policymaking that regulates such marketing. However, before such a registry could be created, there would need to be widely accepted standards for concluding that a marketing practice contributed to premature deaths. In our view, experimental evidence would be needed.

### Experimental Evidence of the Impact of Marketing

Research and litigation (Biglan, 2004) on the harm of cigarette marketing suggest general standards for concluding that marketing is harmful to health (Biglan et al., 2019). In our view, there must be experimental evidence that this marketing contributes to the use of a product that causes premature death. In the USA vs Philip Morris et al., the tobacco industry argued that the correlation between adolescents’ exposure to cigarette advertising and their subsequent smoking was simply due to a pre-existing interest in cigarettes. However, experimental studies that randomized young people to be exposed to marketing, while others were not, showed that exposure to marketing increased favorable attitudes toward smoking and intentions to take up smoking, two well-established predictors of subsequent smoking.

### Making Public Health the Criterion for the Regulation of Marketing

Each of the marketing practices we identify has been opposed by groups seeking to reduce these harms. But these efforts have had limited success because of the widespread belief that the regulation of industry is harmful (Lencucha & Thow, 2019). Overcoming this default assumption is essential for increasing our ability to prevent the premature deaths that result from harmful marketing. That assumption has been promoted by advocates for free market economics who cite Adam Smith’s description of the benefit of unfettered markets. However, their advocacy ignores the fact that Smith actually favored the need for government to restrain harmful commercial practices (Steele et al., 2021).

We argue that the alternative principle should be the public health principle: regulation of practices that contribute to premature death. Economists and the public health community are quite capable of assessing the harms of products to public health. The principle we would advocate is that when the harm of marketing a product exceeds its benefit, that marketing should be subject to regulation to ensure that the marketing is not profitable. This principle would supplant the widely accepted principle that regulation of business should be limited.

Government efforts to combat harmful marketing have generally failed because they rely on fines that are merely a cost of doing business. In 2007, for example, three executives at Purdue Pharma paid $634.5 million in fines for the fraudulent marketing of OxyContin. Yet, the company continued to market the drug quite profitably and overdose deaths continued to rise. The practices of corporations are selected by their consequences. Companies continue to engage in harmful marketing because it is profitable. They will discontinue practices that are losing them money, but fines that are less than the total profits are simply the cost of doing business. Harmful marketing will stop when it is unprofitable.

### Reducing the Power of Corporations

The power of corporations to profit from harmful practices goes well beyond their ability to market harmful products with impunity (Biglan, 2024). For this reason, the problem of reducing harmful marketing needs to be seen in the context of the power that corporations have attained over the past forty years (Biglan, 2020a). In their pursuit of maximizing profits, they have eroded the enforcement of anti-trust laws, gained the ability to fund political candidates, reduced the regulatory authority of government agencies, and learned to influence public opinion through disinformation campaigns. The most recent example of corporate power to thwart regulation is the Supreme Court ruling in Loper Bright Enterprises v. Raimondo, which gave the courts the power to overturn government agency regulations (Furlow, 2024).

Wood et al. (2023) provide an extensive review of the strategies that have been reported for reducing corporate power. Supplemental Table 2 lists the strategies they identified and provides two examples of each strategy. Strengthening these efforts will contribute not only to our ability to prevent harmful marketing, but to the more general effort to prevent harmful corporate practices.

## Supplementary Information

Below is the link to the electronic supplementary material.

Supplementary file1 (DOCX 26 KB)

Supplementary file2 (DOCX 27 KB)
