# Supplementary material for: Harmful Marketing: An Overlooked Social Determinant of Health
Source: Prev Sci. 2025 Jan 10;26(1):138–48. doi: 10.1007/s11121-024-01763-x (PMC11811470; doi:10.1007/s11121-024-01763-x)
Supplement: Supplementary file 1 — Supplementary file1 (DOCX 26 KB) [file 11121_2024_1763_MOESM1_ESM.docx]

**Supplemental Table 1**

*Annual Deaths and Disproportionate Impact on Lower Income, Minoritized Communities in the United States Due to Cigarettes, Alcohol, Processed Food, Opioids, Firearms, and Fossil Fuels*

| Product | Estimate of Annual Deaths | Disproportionate Impact on Lower Income, Minoritized Communities |
| --- | --- | --- |
| Cigarettes | 480,000 (U.S. Department of Health and Human Services et al., 2014) | Tobacco industry marketing specifically targets minoritized populations (Heley et al., 2023; Kingsbury et al., 2020; Lee et al., 2015; National Cancer Institute, 2008; Perks et al., 2018) and smoking disproportionally impacts lower income, less educated, and minoritized populations, including members of the Lesbian, Gay, and Bisexual community (Centers for Disease Control and Prevention, 2023; Mehta et al., 2015; Nandi et al., 2014). |
| Alcohol | 178,307 in 2020-2021 (Esser, 2024) | Minoritized youth are exposed to more alcohol marketing than white youth (Alaniz & Wilkes, 1998; D'Amico et al., 2017), and minoritized and lower income groups are disproportionately impacted by alcohol use, both by health effects and punitive outcomes (Caetano et al., 2014; Clark et al., 2013; Naimi et al., 2008; Zemore et al., 2018). |
| Opioids | Estimated overdose deaths from opioids was 75,673 in the 12-month period ending in April 2021 (Centers for Disease Control and Prevention, 2021), increasing to 107,941 in 2022 (National Institute on Drug Abuse, 2024). Not all these deaths are due to opioids marketed by pharmaceutical companies. However, many of the drug overdose deaths due to illicit drugs are among people who initially became addicted to prescription opioids (Compton et al., 2016). | The health impact of opioids has disproportionately affected rural and ethnic populations, possibly due to lack of access to alternative treatments for pain and addiction treatment (Palombi et al., 2018; Siddiqui & Urman, 2022), as well as more opioids being prescribed in rural areas (Prunuske et al., 2014). |
| Processed Food | 318,656 cardiometabolic deaths associated with dietary factors in 2012 (Micha et al., 2017) | Health risks, including obesity, from unhealthy foods are associated with disproportionate community access to healthy food, with lower income neighborhoods not having adequate access to fruits and vegetables while simultaneously having outsized access to fast-food options (Cooksey-Stowers et al., 2017). Non-Hispanic Black adults have the highest prevalence of obesity (38.4%), followed by Hispanic adults (32.6%) and non-Hispanic white adults (28.6%) (Peterson et al., 2019). Obesity has been linked to increased risk of heart disease (Jensen et al., 2014), type two diabetes (Lu et al., 2013), and cancer (Avgerinos et al., 2019; Bhaskaran et al., 2014) |
| Firearms | 48,204 in 2022, up from 39,740 in 2018 (Centers for Disease Control and Prevention & Statistics, 2024) | In 2021, among males, Black males had the highest age-adjusted rate of firearm-related homicide (52.9 deaths per 100,000 standard population), and among females, Black females had the highest rate (7.5) (Garnett & Spencer, 2023). An analysis of the nearly 68,000 gun-related deaths among people aged 5-24 between 2007 and 2016 showed the risk of dying by firearm was more than double for children and young adults in the poorest counties compared to those living in the richest counties (Barrett et al., 2021). |
| Fossil Fuels | 350,000 premature deaths attributed to fossil fuel pollution (Vohra et al., 2021). Weather-related deaths have risen 35% since 2017; in 2021, there were 974 such deaths (Mailloux et al., 2022). | The public health impact of fossil fuel use and climate change is disproportionately high for minoritized and lower income communities (Donaghy et al., 2023). In one example, due to the presence of chemical plants and oil refineries in one geographic location, the cancer risk among Black and low-income people in Louisiana is nearly 50 times higher than the national average (University Network for Human Rights, 2019). |

References Cited

Alaniz, M. L., & Wilkes, C. (1998). Pro-drinking messages and message environments for young adults: The case of alcohol industry advertising in African American, Latino, and Native American communities. *Journal of Public Health Policy*, *19*(4), 447-472.

Avgerinos, K. I., Spyrou, N., Mantzoros, C. S., & Dalamaga, M. (2019). Obesity and cancer risk: Emerging biological mechanisms and perspectives. *Metabolism*, *92*, 121-135.

Barrett, J. T., Lee, L. K., Monuteaux, M., Hoffmann, J. A., & Fleegler, E. W. (2021). County-Level Poverty and Disparities in Firearm-Related Mortality in US Youth 5-24 Years Old. *Pediatrics*, *147*(3_MeetingAbstract), 503-504.

Bhaskaran, K., Douglas, I., Forbes, H., dos-Santos-Silva, I., Leon, D. A., & Smeeth, L. (2014). Body-mass index and risk of 22 specific cancers: a population-based cohort study of 5· 24 million UK adults. *The Lancet*, *384*(9945), 755-765.

Caetano, R., Vaeth, P. A., Chartier, K. G., & Mills, B. A. (2014). Epidemiology of drinking, alcohol use disorders, and related problems in US ethnic minority groups. *Handb Clin Neurol*, *125*, 629-648. https://doi.org/10.1016/b978-0-444-62619-6.00037-9

Centers for Disease Control and Prevention. (2021). *Drug Overdose Deaths in the U.S. Top 100,000 Annually*. National Center for Health Statistics. Retrieved November 30, 2022 from https://www.cdc.gov/nchs/pressroom/nchs_press_releases/2021/20211117.htm

Centers for Disease Control and Prevention. (2023). *Burden of Cigarette Use in the U.S.: Current Cigarette Smoking Among U.S. Adults Aged 18 Years and Older*. Center for Disease Control and Prevention,. Retrieved August 21, 2024 from https://www.cdc.gov/tobacco/campaign/tips/resources/data/cigarette-smoking-in-united-states.html

Centers for Disease Control and Prevention, & Statistics, N. C. f. H. (2024). *National Vital Statistics System, Mortality 2018-2022 on CDC WONDER Online Database, released in 2024. Data are from the Multiple Cause of Death Files, 2018-2022, as compiled from data provided by the 57 vital statistics jurisdictions through the Vital Statistics Cooperative Program*. Centers for Disease Control and Prevention. Retrieved August 21, 2024 from http://wonder.cdc.gov/ucd-icd10-expanded.html

Clark, T. T., Nguyen, A. B., & Kropko, J. (2013). Epidemiology of drug use among biracial/ethnic youth and young adults: results from a U.S. population-based survey. *J Psychoactive Drugs*, *45*(2), 99-111. https://doi.org/10.1080/02791072.2013.785804

Compton, W. M., Jones, C. M., & Baldwin, G. T. (2016). Relationship between Nonmedical Prescription-Opioid Use and Heroin Use. *New England Journal of Medicine*, *374*(2), 154-163. https://doi.org/10.1056/NEJMra1508490

Cooksey-Stowers, K., Schwartz, M. B., & Brownell, K. D. (2017). Food Swamps Predict Obesity Rates Better Than Food Deserts in the United States. *Int J Environ Res Public Health*, *14*(11). https://doi.org/10.3390/ijerph14111366

D'Amico, E. J., Martino, S. C., Collins, R. L., Shadel, W. G., Tolpadi, A., Kovalchik, S., & Becker, K. M. (2017). Factors associated with younger adolescents' exposure to online alcohol advertising. *Psychol Addict Behav*, *31*(2), 212-219. https://doi.org/10.1037/adb0000224

Donaghy, T. Q., Healy, N., Jiang, C. Y., & Battle, C. P. (2023). Fossil fuel racism in the United States: How phasing out coal, oil, and gas can protect communities. *Energy Research & Social Science*, *100*. https://doi.org/https://doi.org/10.1016/j.erss.2023.103104

Esser, M. B. (2024). Deaths from excessive alcohol use—United States, 2016–2021. *Morbidity and Mortality Weekly Report*, *73*.

Garnett, M. F., & Spencer, M. R. (2023). Age-Adjusted Rates of Firearm-Related Homicide, by Race, Hispanic Origin, and Sex--National Vital Statistics System, United States, 2021. *Morbidity and Mortality Weekly Report*, *72*(6), 737-738.

Heley, K., Popova, L., Moran, M. B., Ben Taleb, Z., Hart, J. L., Wackowski, O. A., Westling, E., Smiley, S. L., & Stanton, C. A. (2023). Targeted tobacco marketing in 2020: the case of #BlackLivesMatter. *Tob Control*, *32*(4), 530-533. https://doi.org/10.1136/tobaccocontrol-2021-056838

Jensen, M., Ryan, D., Donato, K., Apovian, C., Ard, J., Comuzzie, A., Hu, F., Hubbard, V., Jakicic, J., & Kushner, R. (2014). Guidelines 2013 for managing overweight and obesity in adults. Obesity. 2014; 22: S1–S410. *National Institute for Health and Care Excellence (UK). National Institute for Health and Clinical Excellence: guidance. Obesity: identification, assessment and management of overweight and obesity in children, young people and adults: partial update of CG43. National Clinical Guideline Centre (UK)*.

Kingsbury, J. H., D'Silva, J., O'Gara, E., Parks, M. J., & Boyle, R. G. (2020). How Much Progress Have We Made? Trends in Disparities in Tobacco Use. *Prev Chronic Dis*, *17*, E107. https://doi.org/10.5888/pcd17.200090

Lee, J. G., Henriksen, L., Rose, S. W., Moreland-Russell, S., & Ribisl, K. M. (2015). A Systematic Review of Neighborhood Disparities in Point-of-Sale Tobacco Marketing. *Am J Public Health*, *105*(9), e8-18. https://doi.org/10.2105/ajph.2015.302777

Lu, Y., Hajifathalian, K., Ezzati, M., Woodward, M., Rimm, E. B., & Danaei, G. (2013). Metabolic mediators of the effects of body-mass index, overweight, and obesity on coronary heart disease and stroke: a pooled analysis of 97 prospective cohorts with 1· 8 million participants. *Lancet (London, England)*, *383*(9921), 970-983.

Mailloux, N. A., Abel, D. W., Holloway, T., & Patz, J. A. (2022). Nationwide and Regional PM2.5-Related Air Quality Health Benefits From the Removal of Energy-Related Emissions in the United States. *Geohealth*, *6*(5), e2022GH000603. https://doi.org/https://doi.org/10.1029/2022GH000603

Mehta, N. K., House, J. S., & Elliott, M. R. (2015). Dynamics of health behaviours and socioeconomic differences in mortality in the USA. *J Epidemiol Community Health*, *69*(5), 416-422. https://doi.org/10.1136/jech-2014-204248

Micha, R., Peñalvo, J. L., Cudhea, F., Imamura, F., Rehm, C. D., & Mozaffarian, D. (2017). Association Between Dietary Factors and Mortality From Heart Disease, Stroke, and Type 2 Diabetes in the United States. *JAMA*, *317*(9), 912-924. https://doi.org/10.1001/jama.2017.0947

Naimi, T. S., Cobb, N., Boyd, D., Jarman, D. W., Brewer, R., Nelson, D. E., Holt, J., Espey, D., Snesrud, P., & Chavez, P. (2008). Alcohol-attributable deaths and years of potential life lost among American Indians and Alaska Natives--United States, 2001--2005. *MMWR: Morbidity and Mortality Weekly Report*, *57*(31), 938-941.

Nandi, A., Glymour, M. M., & Subramanian, S. V. (2014). Association among socioeconomic status, health behaviors, and all-cause mortality in the United States. *Epidemiology*, *25*(2), 170-177. https://doi.org/10.1097/ede.0000000000000038

National Cancer Institute. (2008). *The role of the media in promoting and reducing tobacco use. Tobacco Control Monograph No. 19* (R. M. Davis, E. A. Gilpin, B. Loken, K. Viswanath, & M. A. Wakefield, Eds. Vol. NIH Pub. No. 07-6242). U.S. Department of Health and Human Services, National Institutes of Health, National Cancer Institute.

National Institute on Drug Abuse. (2024). *Drug Overdose Death Rates*. National Institute on Drug Abuse. Retrieved August 21, 2024 from https://nida.nih.gov/research-topics/trends-statistics/overdose-death-rates

Palombi, L. C., St Hill, C. A., Lipsky, M. S., Swanoski, M. T., & Lutfiyya, M. N. (2018). A scoping review of opioid misuse in the rural United States. *Ann Epidemiol*, *28*(9), 641-652. https://doi.org/10.1016/j.annepidem.2018.05.008

Perks, S. N., Armour, B., & Agaku, I. T. (2018). Cigarette Brand Preference and Pro-Tobacco Advertising Among Middle and High School Students - United States, 2012-2016. *Mortality and Morbidity Weekly Report*, *67*(4), 119-124. https://neuro.unboundmedicine.com/medline/citation/29389916/Cigarette_Brand_Preference_and_Pro_Tobacco_Advertising_Among_Middle_and_High_School_Students___United_States_2012_2016_https://doi.org/10.15585/mmwr.mm6704a3

Peterson, B., Cramer, L., Fontaine, J., & Urban Institute. (2019). Policies and practices for children of incarcerated parents: Summarizing what we know and do not know. In J. M. Eddy & J. Poehlmann (Eds.), *Handbook on children with incarcerated parents: Research, policy, and practice*. Springer International Publishing. https://doi.org/10.1007/978-3-030-16707-3

Prunuske, J. P., St Hill, C. A., Hager, K. D., Lemieux, A. M., Swanoski, M. T., Anderson, G. W., & Lutfiyya, M. N. (2014). Opioid prescribing patterns for non-malignant chronic pain for rural versus non-rural US adults: a population-based study using 2010 NAMCS data. *BMC Health Serv Res*, *14*, 563. https://doi.org/10.1186/s12913-014-0563-8

Siddiqui, N., & Urman, R. D. (2022). Opioid Use Disorder and Racial/Ethnic Health Disparities: Prevention and Management. *Curr Pain Headache Rep*, *26*(2), 129-137. https://doi.org/10.1007/s11916-022-01010-4

U.S. Department of Health and Human Services, Centers for Disease Control and Prevention, National Center for Chronic Disease Prevention and Health Promotion, & Office on Smoking and Health. (2014). *The health consequences of smoking: 50 years of progress. A Report of the Surgeon General*. U.S. Department of Health and HumanServices, Centers for Disease Control and Prevention, National Center for Chronic Disease Prevention and Health Promotion, Office on Smoking and Health.

University Network for Human Rights. (2019). *Waiting to Die: Toxic Emissions and Disease Near the Louisiana Denka/DuPont Plant*. University Network for Human Rights. http://www.humanrightsnetwork.org/waiting-to-die

Vohra, K., Vodonos, A., Schwartz, J., Marais, E. A., Sulprizio, M. P., & Mickley, L. J. (2021). Global mortality from outdoor fine particle pollution generated by fossil fuel combustion: Results from GEOS-Chem. *Environ Res*, *195*, 110754. https://doi.org/10.1016/j.envres.2021.110754

Zemore, S. E., Karriker-Jaffe, K. J., Mulia, N., Kerr, W. C., Ehlers, C. L., Cook, W. K., Martinez, P., Lui, C., & Greenfield, T. K. (2018). The Future of Research on Alcohol-Related Disparities Across U.S. Racial/Ethnic Groups: A Plan of Attack. *J Stud Alcohol Drugs*, *79*(1), 7-21. https://doi.org/10.15288/jsad.2018.79.7
