# Supplementary material for: Harmful Marketing: An Overlooked Social Determinant of Health
Source: Prev Sci. 2025 Jan 10;26(1):138–48. doi: 10.1007/s11121-024-01763-x (PMC11811470; doi:10.1007/s11121-024-01763-x)
Supplement: Supplementary file 2 — Supplementary file2 (DOCX 27 KB) [file 11121_2024_1763_MOESM2_ESM.docx]

**Supplemental Table 2**

*Strategic Objectives, Illustrative Examples of Actions to Curb Excessive Power, Adapted from Wood et al. (Wood et al., 2023) and Meagher (Meagher, 2020a, 2020b).*

| Strategic Objective | Strategy | Examples of Action |
| --- | --- | --- |
| Disperse concentrated corporate wealth and power | - Strengthen antitrust regulation - Strengthen regulation of political contributions | - Widen objectives of antitrust policy to consider broader welfare concerns - Regulate corporate contributions to political candidates and parties |
| Strengthen countervailing power structures | - Strengthen the countervailing power of workers and consumers - Support legal remediation for citizens harmed by corporations - Organize alternative modes of business and systems of production and distribution | - Strengthen labor and unionization laws - Support the use of *qui tam* suits by citizens against corporations - Scale-up alternative forms of enterprise, such as worker co-operatives and mutual enterprise |
| Democratize corporate decision-making | - Improve stakeholder representation on corporate boards - Mandate corporate decision-makers to identify and mitigate adverse social and environmental impacts | - Mandate stakeholder representation requirements on corporate boards - Implement robust corporate due diligence laws that consider human rights and environmental sustainability |
| Reform and democratize the global governance of corporations | - Reform and democratize existing international organizations and institutional arrangements that sustain corporate power - Develop new international organizations and institutional arrangements that constrain corporate power | - Assign a greater role to national governments in the negotiation and ratification of WTO agreements - Revive plans to develop global institutions to govern transnational corporations |
| Dissolve excessive and harmful corporate power | - Wind-down harmful industries - Reform/transform the corporate form | - Scale-up industrial policy that drives systematic transition from non-renewable to renewable energy sources - Revoke limited liability for all corporations above a certain size in terms of assets or revenue |

*Note.* Abbreviation: WTO, World Trade Organization.

**References**

Meagher, M. (2020a). *Competition is killing us: how big business is harming our society and planet-and what to do about it*. Penguin Business.

Meagher, M. (2020b). Winner takes all. *Royal Society for Arts*, *166*(3), 32-34. <https://www.thersa.org/comment/2021/01/journal-winner-takes-all>

Wood, B., Lacy-Nichols, J., & Sacks, G. (2023). Taking on the Corporate Determinants of Ill-health and Health Inequity: A Scoping Review of Actions to Address Excessive Corporate Power to Protect and Promote the Public’s Health. *International Journal of Health Policy and Management*, *12*(Issue 1), 1-17. <https://doi.org/10.34172/ijhpm.2023.7304>
